# Severe Erythromelalgia Pain Attack in a Young Lebanese Woman Leading to Hospitalization: A Case Report and Literature Review

**DOI:** 10.7759/cureus.91530

**Published:** 2025-09-03

**Authors:** Wassim Assaad, Omar El Tarras, Soad Al Osta, Chady Kallassy

**Affiliations:** 1 Internal Medicine, Lebanese American University Medical Center–Rizk Hospital, Beirut, LBN; 2 Vascular Medicine, Lebanese American University Medical Center–Rizk Hospital, Beirut, LBN

**Keywords:** cellulitis, erythromelalgia, multidisciplinary care, neurovascular syndrome, pain

## Abstract

Erythromelalgia is a rare neurovascular syndrome characterized by intense, episodic burning pain that primarily affects the feet and hands and, occasionally, the face. Symptoms are often triggered by heat and exercise, with relief typically achieved through cooling methods. However, improper use of these techniques can lead to serious complications, such as trench foot and cellulitis. The condition can also have significant psychological effects, contributing to anxiety and depression. An 18-year-old Lebanese woman with primary erythromelalgia and a family history of the condition presented with worsening bilateral pain and erythema in her lower extremities. Her pain was poorly controlled, prompting her to engage in prolonged ice-water immersion, which resulted in skin abrasions, necrotic ulcers, and cellulitis. On admission, she exhibited bilateral lower extremity erythema, edema, tenderness, and macerated skin with necrotic ulcers on her left foot. Her nails showed white discoloration and onycholysis. Laboratory tests were normal, and Doppler ultrasound revealed increased blood flow, supporting the diagnosis of erythromelalgia. A multidisciplinary team managed her care, addressing infection, pain, and anxiety. She received antibiotics, wound care, and antifungal therapy for onychomycosis. Pain management included aspirin, pregabalin, topical lidocaine, acetaminophen, nefopam, and opiates; however, due to persistent pain, her regimen was adjusted to incorporate morphine and additional agents. Duloxetine was also introduced to address both anxiety and pain. After two days on the revised treatment plan, her pain improved significantly, allowing for discharge. Follow-up visits confirmed skin healing, and Doppler ultrasound again demonstrated increased blood flow. This case highlights the complexity of managing severe erythromelalgia, underscoring the importance of appropriate pain management, patient education, and multidisciplinary care. It represents the first reported case of erythromelalgia requiring hospitalization in Lebanon and illustrates the potential complications of inadequate management and inappropriate use of cooling techniques.

## Introduction

Erythromelalgia is a rare clinical neurovascular syndrome characterized by severe, episodic burning sensations that primarily affect the feet, followed by the hands and face [1,2]. Symptoms are often triggered by exercise and heat exposure, and relief is typically achieved through cooling techniques such as cold/ice water immersion and fanning [3]. However, inappropriate use of these methods can result in complications, including trench foot and cellulitis [4].

In addition to its physical manifestations, erythromelalgia can significantly impair quality of life and may contribute to depression and anxiety. In a study of 168 patients, Friberg et al. reported that individuals with erythromelalgia had significantly lower mental and physical health scores compared to the general population, as measured by the SF-36 survey [5]. Many participants struggled with pain management, mobility limitations, and life-altering symptoms, with some ultimately requiring wheelchairs [5].

This case report presents the first documented instance of severe erythromelalgia in Lebanon requiring hospitalization due to intractable pain and complications, including cellulitis. It underscores the importance of a multidisciplinary approach to patient management and education and is accompanied by a review of the literature.

## Case presentation

An 18-year-old Lebanese woman with a history of primary erythromelalgia and a paternal family history of the condition presented with worsening pain and erythema in both lower extremities, unresponsive to outpatient pain management (Table 1).

**Table 1:** Summary of the patient’s treatment prior to presentation, during hospitalization, and at discharge. NSAIDs, nonsteroidal anti-inflammatory drugs

Treatment site	Day of treatment	Medications received	Notes
Home	-	Acetaminophen	
		Codeine	
		Gabapentin	
		Tramadol	As needed
During hospitalization	Day 1	Aspirin	
		Opiates	Meperidine or morphine, depending on availability
		Nefopam	
		Lidocaine gel	
		Pregabalin	
		Duloxetine	
		Amoxicillin-clavulanic acid	
	Day 2	NSAIDs added	
	Day 3	Magnesium supplements	
		Carbamazepine	
		Bisoprolol	
		Cetirizine	
		Discontinued lidocaine gel	Due to lack of benefit
	Day 4	Acetaminophen resumed	Initially stopped due to lack of benefit
		Discontinued opiates and nefopam	
At home after discharge	-	Aspirin	
		Paracetamol + codeine formulation	As needed
		Pregabalin	
		Carbamazepine	
		Duloxetine	
		Magnesium supplements	
		Bisoprolol	

**Figure 1:**
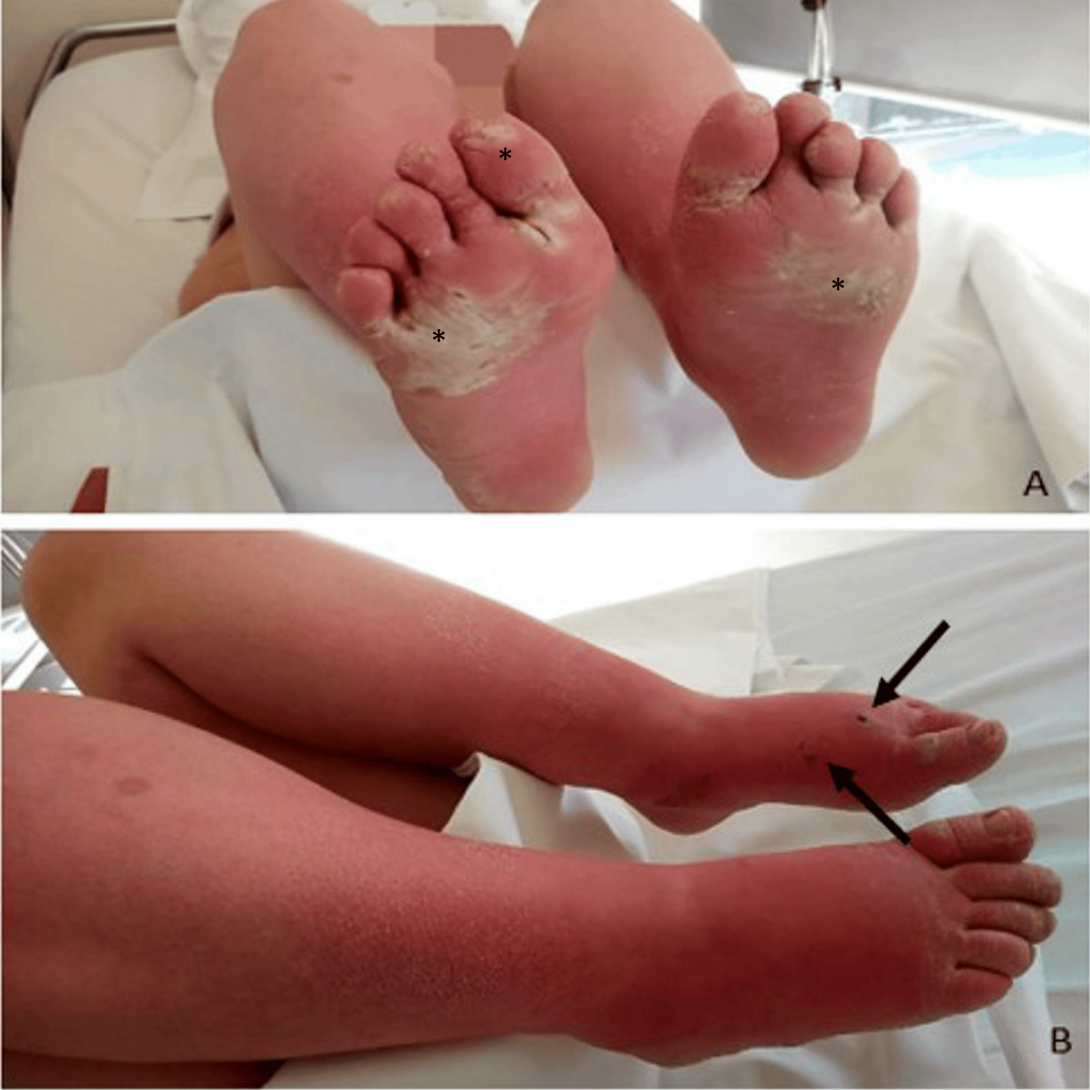
Skin findings in the erythromelalgia patient upon presentation to the emergency department for severe pain. Marked erythema of both feet and shins with diffuse edema was observed. Skin exfoliation and abrasions (*) were noted (A and B). Necrotic skin lesions of the left foot are indicated by black arrows.

The pain had persisted for two months, and during the two weeks preceding her presentation, she immersed her legs in ice water for prolonged periods, which resulted in skin abrasions, necrotic ulcers, and signs of cellulitis. She reported that her symptom exacerbations were typically more pronounced during late spring and summer; however, the index presentation represented the most severe episode she had experienced to date. Upon admission, the patient exhibited bilateral erythema, subcutaneous edema, tenderness, and macerated skin with necrotic ulcers on her left foot (Figure 1).

**Figure 2:**
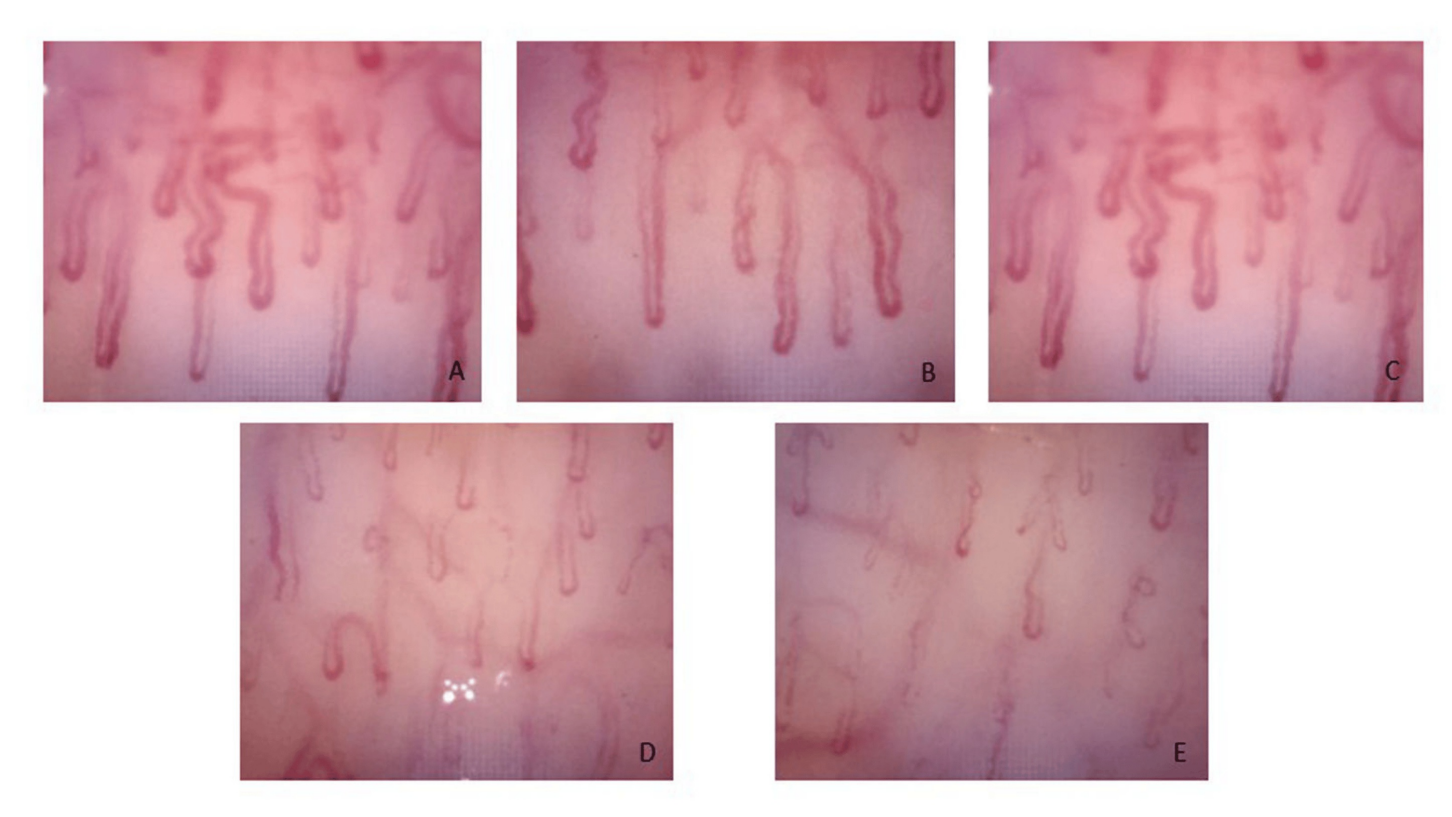
Capillaroscopy findings in the patient with erythromelalgia. (A-C) Hyperemia due to dilated venular and arteriolar segments of the capillaries, with increased flow volume producing reddish capillaries. (D, E) Presence of precapillary shunts, with decreased capillary circulation as flow is diverted into the subpapillary and reticular venous plexus. These findings are thought to be related to repetitive ice and cold-water immersion, leading to contraction of the precapillary sphincters.

The toenails of both feet showed leukonychia, mild onychauxis, and onycholysis (Figure 2).

**Figure 3:**
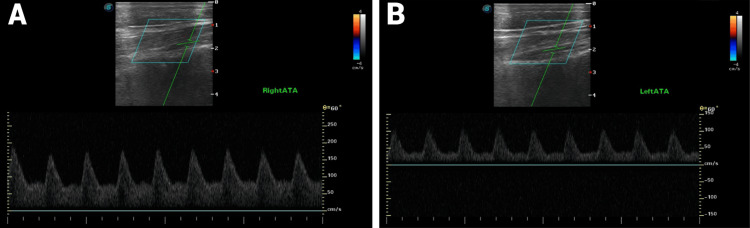
Doppler ultrasound findings at presentation. Accelerated systolic-diastolic waveforms at the anterior tibial arteries suggest hyperemia and capillary shunting (A, B). Increased flow volume was measured at the superficial femoral artery (825 mL/min). The potential consequences on cardiac output should be further assessed by echocardiography.

Laboratory results showed normal CBC, electrolytes, fasting blood glucose, antinuclear antibody, and thyroid-stimulating hormone. Doppler ultrasound of the lower extremities demonstrated increased blood flow and high blood volume in the lower limbs, findings suggestive of erythromelalgia. No features of peripheral arterial disease or arteriopathy were observed (Figure 3).

Echocardiography, performed concurrently with Doppler ultrasound of the lower limbs, showed a normal ejection fraction greater than 55% and a cardiac output of 3.75 L/min/m², without evidence of pulmonary hypertension. Differential diagnoses included cellulitis secondary to inappropriate cold-water immersion, erythromelalgia exacerbation, and second-degree burns.

The patient was initially treated for cellulitis with amoxicillin-clavulanic acid (1 g twice daily for 10 days), along with wound care. For concurrent onychomycosis, she was prescribed terbinafine (250 mg daily for eight weeks). To manage her pain, she received aspirin (300 mg daily), pregabalin (30 mg daily), topical lidocaine gel, acetaminophen, and nefopam as needed. Intermittent cooling with a fan was also permitted. Despite these interventions, her pain remained significant, necessitating multiple doses of morphine for adequate control.

Due to persistent symptoms, her regimen was adjusted to include carbamazepine (100 mg twice daily), celecoxib (200 mg daily), and oxycodone (5 mg three times daily), titrated according to her opioid requirements. She was also started on bisoprolol (2.5 mg daily) and cetirizine (10 mg twice daily) in addition to her previous treatments. The psychiatry team evaluated her for anxiety and initiated duloxetine (30 mg daily) to further optimize pain control.

Within two days of implementing the revised treatment plan, which included carbamazepine, celecoxib, oxycodone, bisoprolol, cetirizine, and duloxetine, the patient experienced significant pain relief without adverse events. With resolution of the erythema in her legs and improved pain control, she was discharged from the hospital with compression stockings, a revised pain management regimen, and wound care follow-up (Table 1).

**Figure 4:**
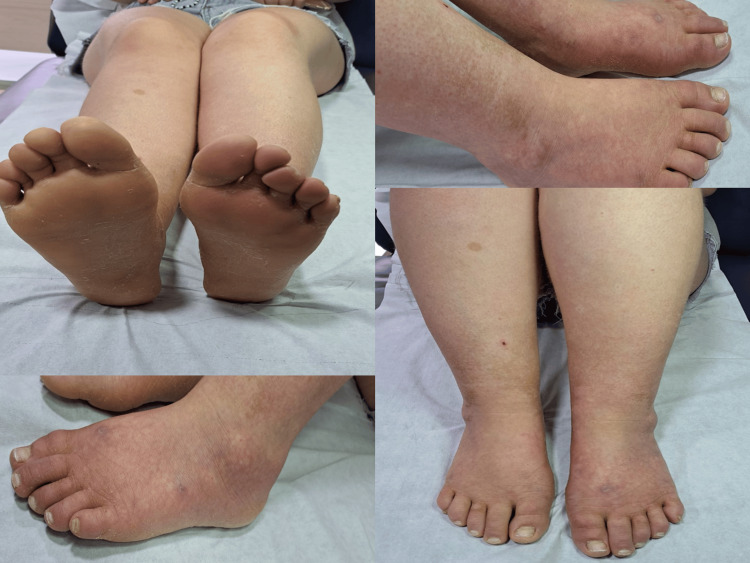
Physical examination findings after several months of treatment. Mild edema of the lower extremities persists. Ongoing healing of the necrotic skin ulcer is observed on the left foot, and the nails are free of onychomycosis. Erythema has resolved and been replaced by mild cyanosis, with post-inflammatory hyperpigmentation following months of skin inflammation.

**Figure 5:**
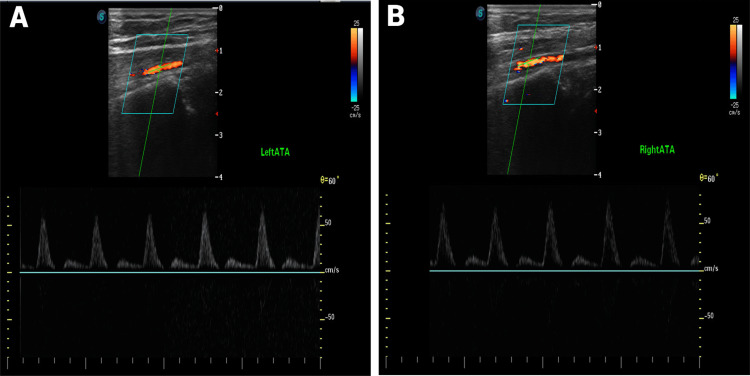
Doppler ultrasound findings after several months of treatment (A, B). A normal, nonaccelerated triphasic waveform is observed in both anterior tibial arteries, with normal heart rate and flow volume.

Over the subsequent two weeks, her condition continued to improve, allowing for discontinuation of cooling techniques, opioids, and nonsteroidal anti-inflammatory drugs (NSAIDs). Physical examination revealed marked skin healing (Figure 4), and follow-up Doppler ultrasounds performed two weeks and several months after discharge demonstrated normal triphasic distal flow (Figure 5).

## Discussion

Erythromelalgia, first described by Mitchell in 1878, is a rare neurovascular syndrome characterized by intermittent burning pain and erythema that primarily affects the extremities, most commonly the lower limbs, followed by the hands and, less frequently, the ears and face [1,4]. Symptoms are typically exacerbated by heat, physical activity, and ambulation and are relieved by cooling and elevation [3,6]. The rarity of erythromelalgia complicates the establishment of comprehensive epidemiological data, often leading to underdiagnosis due to its broad clinical spectrum and the absence of specific diagnostic tests. Reported age at presentation ranges widely, from five to 90 years, with a mean age of approximately 55 years in an Olmsted study and seven to 76 years in a Norwegian case series [7,8]. Females are affected more frequently than males [7,8].

Erythromelalgia is classified into primary and secondary forms based on etiology (Table 2).

**Table 2:** Causes of secondary erythromelalgia.

Category	Conditions
Myeloproliferative diseases	Essential thrombocythemia
	Myelodysplastic syndrome
	Polycythemia vera
	Leukemia
Neoplasia	Primary colon cancer
	Breast cancer
	Thymoma
	Astrocytoma
	Paraneoplastic syndrome
	Subcutaneous panniculitis
Connective tissue disorders	Systemic lupus erythematosus
	Rheumatoid arthritis
	Vasculitis
	Sjögren syndrome
Metabolic diseases	Diabetes mellitus types 1 and 2
	Gout
	Hypercholesterolemia
Neuropathies	Multiple sclerosis
	Small fiber neuropathies
	Neurofibromatosis
Drugs	Influenza and hepatitis B vaccines
	Calcium channel blockers
	Bromocriptine
	Cyclosporine
Other	Mushroom intoxication
	Mercury poisoning
	Lichen sclerosus
	Burns and frostbite

Primary erythromelalgia, also referred to as inherited or idiopathic erythromelalgia, is linked to mutations in the SCN9A gene, inherited in an autosomal dominant manner [3,6,9]. These mutations affect sympathetic and nociceptive neurons, resulting in microvascular symptoms and severe burning pain. Specifically, SCN9A mutations produce gain-of-function changes in the Nav1.7 sodium channel, leading to neuronal hyperexcitability and reduced activation thresholds in sensory and sympathetic neurons, which underlie the hallmark burning pain of primary erythromelalgia [6,9]. Secondary erythromelalgia is associated with other medical conditions, most notably myeloproliferative disorders, in which platelet activation and thrombosis contribute to the pathogenesis through prostaglandin-mediated coagulation pathways [3].

Diagnosis relies heavily on clinical history and physical examination, typically characterized by burning pain, erythema, and warmth of the skin, with symptom relief upon cooling. Ancillary tests such as thermography may demonstrate increased skin temperature, while skin biopsies can reveal decreased nerve density or intraluminal thrombi [3,10]. Genetic testing for SCN9A mutations can confirm primary erythromelalgia but is not routinely required [9].

In the presented case, the patient’s pain was refractory to traditional cooling methods, likely due to environmental factors such as rising temperatures and humidity, compounded by self-inflicted skin injuries from excessive cold-water immersion. Liu et al. demonstrated that wide temperature fluctuations between winter and early spring were associated with increased erythromelalgia epidemics in China [11]. Although this case occurred in Lebanon, the patient reported symptom exacerbations during periods of sudden temperature shifts and increased humidity, mirroring the environmental pattern described by Liu et al. Repeated exposure to cold water and fanning can disrupt the skin barrier, resulting in maceration, ulceration, and secondary infections. These injuries contribute to peripheral sensitization by exposing nociceptors to inflammatory mediators and damaged tissue, thereby amplifying neuropathic pain in patients with erythromelalgia [12,13]. As pain worsens, patients often engage in a vicious cycle of cooling, skin injury, and further pain, underscoring the dangers of maladaptive coping strategies in erythromelalgia.

Management of erythromelalgia remains challenging due to the limited understanding of its pathophysiology and the absence of standardized treatment guidelines. A wide range of therapeutic approaches has been described in the literature. No single therapy has demonstrated consistent efficacy, with treatment responses varying not only among different patients but also within the same individual over time. This variability highlights the unpredictable and complex nature of the disease. Current strategies focus on trigger avoidance, pharmacotherapy, and, in refractory cases, procedural interventions (Table 3) [12].

**Table 3:** Summary of behavioral, pharmacological, nonpharmacological, and procedural interventions reported in the literature for the treatment of patients with erythromelalgia. CCBs, calcium channel blockers; NSAIDs, nonsteroidal anti-inflammatory drugs; SNRIs, serotonin-norepinephrine reuptake inhibitors; SSRIs, selective serotonin reuptake inhibitors

Therapy type	Interventions
Behavioral therapies	Avoid hot environments
	Avoid exercise
	Avoid wearing excessive clothing
	Leg elevation
Topical therapies (gel, cream, or patches)	Available for several drug classes, including lidocaine, NSAIDs, ketamine, gabapentin, midodrine, and capsaicin formulations
Systemic therapies	Aspirin
	NSAIDs (IV and oral formulations)
	SSRIs and SNRIs
	Gabapentin and pregabalin
	Vasodilators: CCBs, sodium nitroprusside, prostaglandins, and prostacyclin
	Sodium channel blockers: lidocaine, mexiletine, carbamazepine
	Corticosteroids
	Antihistamines
	Beta-blockers
	High-dose magnesium
Procedural interventions	Local botulinum toxin injection
	Sympathetic and epidural blockage
	Sympathectomy
	Spinal cord stimulators
Nonpharmacological and rehabilitation	Pain rehabilitation
	Hypnosis
	Massage therapies
	Biofeedback treatment

Pharmacological treatments included aspirin and ticlopidine for cases associated with myeloproliferative disorders [13]. Prostaglandins, prostacyclin analogs (e.g., IV iloprost), calcium channel blockers, and sodium nitroprusside have been used in several cases, with reports of improvement or remission. Their primary mechanism of action is thought to be related to vasodilation, reversing arteriovenous shunting and hypoxia that cause pain [12,14-16]. Opiates were generally reserved for acute pain management due to the risks of addiction and adverse effects [14].

Sodium channel blockers targeting mutated voltage-gated sodium channels have shown efficacy in preclinical studies of hereditary erythromelalgia [17]; however, they remain unavailable in clinical practice. Lidocaine and mexiletine, which are widely used in the treatment of pain and arrhythmias, can partially block sodium-gated channels in sensory neurons, delaying depolarization and reducing pain perception. Several case reports highlighted the role of these drugs in controlling refractory pain [18,19]. Carbamazepine, another sodium channel blocker used in neuropathic pain and epilepsy, has shown preferential responsiveness in patients with familial inherited erythromelalgia carrying specific voltage-gated sodium channel mutations [20-22].

Gabapentinoids (gabapentin, pregabalin) have also been used for neuropathic pain. They are thought to act centrally by modulating L-type voltage-gated calcium channels involved in pain perception, with several case reports reporting successful outcomes [22-24].

Antihistamines have been proposed for the treatment of erythromelalgia due to their ability to alter blood flow and the possibility of a chronic local allergic reaction caused by the disease. In one survey of members of The Erythromelalgia Association, 40% reported marked improvement with first-generation antihistamines [12]. Al-Minshawy and El-Mazary described a case of a 34-month-old child who demonstrated partial responsiveness to cetirizine, suggesting a possible therapeutic role for antihistamines [25].

Beta-blockers have also been reported to be effective in erythromelalgia, although their mechanism of action remains unclear and may relate to reducing vasodilation [12,26,27]. Antidepressants, including serotonin-norepinephrine reuptake inhibitors and selective serotonin reuptake inhibitors, have shown efficacy in modulating sympathetic activity and vascular control [28,29]. Venlafaxine and serotonin-based therapies have been used with variable results. Topical treatments such as lidocaine and capsaicin may provide localized pain relief, though their availability is limited.

Systemic corticosteroid therapy has been described in several cases. In a retrospective study of 31 patients, Pagani-Estévez et al. identified subsets of corticosteroid-responsive patients [30]. Predictors of response included recent trauma, surgery, or infection [30]. A temporal profile also appeared to play a role, with subacute presentations, defined as crescendo to peak intensity in less than 21 days, being more responsive to corticosteroid therapy [30]. High-dose magnesium supplementation, administered orally or intravenously, was reported in an informal study of 13 patients who had failed multiple drug regimens [31].

Topical agents may be used to avoid systemic adverse effects. Preparations containing amitriptyline-ketamine, lidocaine, midodrine (α1-agonist), NSAIDs, and gabapentin have been described, with variable success in symptom control [32,33]. Capsaicin (trans-8-methyl-N-vanillyl-6-nonenamide), derived from chili peppers and commonly used for diabetic and postherpetic neuropathy, has shown success in alleviating pain in erythromelalgia in several case reports [34,35].

Procedural interventions have been employed in refractory cases unresponsive to pharmacological treatment, though reported numbers remain small. Botulinum toxin injection, considered less invasive, acts by blocking substance P and glutamate release to reduce pain [36]. Subcutaneous botulinum injection in a grid-like pattern over affected areas produced pain relief within three days, lasting up to nine weeks [37]. More invasive procedures, such as sympathetic or epidural block with bupivacaine and fentanyl, sympathectomy, or spinal cord stimulation, have also been reported as successful in refractory cases [38-40].

Pain rehabilitation programs have been shown to reduce depression and negative pain perception. Hypnosis, massage therapies, and biofeedback have also been described in several case reports [41,42].

## Conclusions

Erythromelalgia is a rare neurovascular syndrome characterized by episodic erythema and intense burning pain in the face and extremities. The agonizing nature of the pain often drives patients to adopt unsafe cooling practices, which can worsen symptoms and lead to complications such as cellulitis and skin necrosis. This case represents the first reported instance of erythromelalgia in Lebanon requiring hospitalization and highlights two critical aspects of care: the need to optimize pharmacological management and the importance of thorough patient education on safe cooling practices. Furthermore, it underscores the essential role of a coordinated multidisciplinary approach in addressing the complex challenges posed by this condition.
